# Relevance and Recommendations for the Application of Cardioplegic Solutions in Cardiopulmonary Bypass Surgery in Pigs

**DOI:** 10.3390/biomedicines9091279

**Published:** 2021-09-21

**Authors:** Anna Glöckner, Susann Ossmann, Andre Ginther, Jagdip Kang, Michael A. Borger, Alexandro Hoyer, Maja-Theresa Dieterlen

**Affiliations:** University Department for Cardiac Surgery, Heart Center Leipzig, HELIOS Clinic, 04289 Leipzig, Germany; Anna.Gloeckner@gmx.de (A.G.); Susann.Ossmann@helios-gesundheit.de (S.O.); Andre.Ginther@helios-gesundheit.de (A.G.); jagdip.kang@helios-gesundheit.de (J.K.); Michael.Borger@helios-gesundheit.de (M.A.B.); Alexandro.Hoyer@helios-gesundheit.de (A.H.)

**Keywords:** pig model, animal model, cardioplegia, refinement, cardiopulmonary bypass, cardiac surgery

## Abstract

Cardioplegic solutions play a major role in cardiac surgery due to the fact that they create a silent operating field and protect the myocardium against ischemia and reperfusion injury. For studies on cardioplegic solutions, it is important to compare their effects and to have a valid platform for preclinical testing of new cardioplegic solutions and their additives. Due to the strong anatomical and physiological cardiovascular similarities between pigs and humans, porcine models are suitable for investigating the effects of cardioplegic solutions. This review provides an overview of the results of the application of cardioplegic solutions in adult or pediatric pig models over the past 25 years. The advantages, disadvantages, limitations, and refinement strategies of these models are discussed.

## 1. Introduction

Cardioplegic solutions are essential in cardiac surgery since they create a silent operating field and protect the myocardium against extensive ischemic damage and ischemia-reperfusion injury (IRI). Cardioplegia is defined as controlled-induced cardiac arrest [1,2]. A cardioplegic solution induces cardioplegia leading to reversible cardiac arrest. To create a bloodless surgical field, the heart should be excluded from circulation by aortic clamping. This induces whole-organ ischemia of the heart, which can be tolerated for only a few minutes without additional protection [1,2]. The application of a cardioplegic solution increases the time of ischemic tolerance in the heart for up to several hours. Furthermore, cardiopulmonary bypass (CPB) compensates for the pump function of the heart, provides oxygen and nutrients to organs and tissues, and removes metabolites.

The basic principle of any cardioplegic solution is electromechanical decoupling, which influences the extracellular and intracellular ion concentrations. With this, the energy consumption of the myocardium is significantly reduced and ischemia tolerance is increased [2]. In recent years, several different cardioplegic solutions have been developed [3,4], which are based on either a crystalloid electrolyte solution or patients‘ blood with added electrolytes. However, there are no national or international guidelines or recommendations for choosing cardioplegic solutions for different cardiac surgery procedures [5]. Hence, the selection is largely based on the personal preferences of the surgeon. Furthermore, this must be constantly adapted to new conditions.

Due to demographic changes, patients undergoing cardiac surgery are becoming older and sicker, which necessitates complex cardiac procedures, such as combined heart valve/coronary bypass surgery [6]. Cardioplegic solutions must be adapted to this patient population to provide sufficient myocardial protection and to minimize cardiac damage.

## 2. Clinical Relevance of Data Analyzing Cardioplegic Solutions in Pig Models

Due to the changing characteristics of the patient cohort and more complex surgical interventions in cardiac patients, it is essential to investigate the effects of cardioplegic solutions. Furthermore, it is necessary to perform structured comparisons and to identify the advantages of different cardioplegic solutions. Especially for new compositions of cardioplegic solutions, adequate tests for their safety and efficiency are necessary. Also, translational research requires testing new drugs in two independent species to fulfill the criteria of the application for ethical and regulatory approval [7].

The pathophysiological processes that are induced by cardioplegic arrest of the heart and CPB are very complex and also affect the kidneys, brain, gut, and lungs. If the effects of cardiac cardioplegia and CPB need to be further investigated, invasive procedures, such as biopsy withdrawal, are necessary. For ethical reasons, it is not possible to conduct studies, including extensive biopsy withdrawal, directly in humans. Hence, animal models are used. Alternative methods to investigate the effects of cardioplegic solutions, such as cell cultures or isolated organs, are not able to fully display the effects of surgical intervention and CPB, such as surgical trauma, blood loss, blood contact with foreign surfaces and shear stress during CPB, inflammatory response to CPB, and changes in the coagulation system [8,9,10]. The animal model is therefore of particular clinical relevance. However, the ethical consideration of the risk-benefit balance in animal experiments is significant. The benefit of information from a study should always be greater than the expected risks and suffering of the animals. Throughout the entire study, the focus must be on animal welfare along with the achieved results. Therefore, good experimental planning is necessary, and the requirements of the study must be precisely defined to achieve satisfactory validity of the results. Owing to the reproducibility of the study, it is important to investigate meaningful parameters in a targeted manner [11,12]. The first step is the selection of a suitable animal model that produces transferable results for future human clinical applications. In many cardiac surgery studies, pig models have been established due to their special anatomical and physiological similarities to the human heart [13]. Thus, not only heart valves and coronary care are comparable, but also the hemodynamics of the circulatory system. Furthermore, the responses to certain events, such as the lack of volume, are very similar in pigs and humans [14,15]. Thus, the results obtained from pig models can be transferred to humans [14].

This review provides an overview of the in vivo application of cardioplegic solutions in adult and pediatric pig models over the past 25 years. This review focuses on the induction of cardioplegic arrest in CPB procedures, except for the preservation strategies necessary for heart transplantation. Investigations in isolated pig hearts and in vitro studies were excluded from the analysis. The advantages, disadvantages, limitations, and refinement strategies of the pig models are discussed.

## 3. Comparability of the Heart Anatomy and Physiology in Pig Models and Humans

The pig model has useful biometric conditions regarding the size and anatomy of the cardiovascular system (heart, atria, aorta, femorales and jugulars, coronary vessels, and coronary sinus) [16]. Furthermore, several physiological and hemodynamic similarities exist between the cardiovascular system of pigs and humans [17]. The receptor profiles, ion channels, sympathoadrenal innervation, coronary circulation, and electrophysiology of the pig heart are comparable to those in humans [18]. A lack of volume or loss of blood induced a comparable response in pigs and humans [18]. In response to cardiac arrest and CPB support, it is necessary that the left ventricle ends at the apex, which simplifies physiological measurements such as pressure-volume loops. The pig heart shows limited collateral blood flow, which is analogous to humans and makes it ideal for ischemia studies [19]. However, there is higher cardiac output in pigs, which results from higher heart rate and stroke volume. Furthermore, both parameters resulted from lower hematocrit and oxygen transport capacity [20]. Despite these similarities, long-term follow-up in pig models is considered problematic [19]. If juvenile animals are utilized, changes in animal weight result in alterations in basic cardiac physiology. For example, the heart/body weight ratio is approximately 5 g/kg in healthy humans and 25–30 kg in juvenile farm pigs. However, this ratio decreases up to 50% in farm pigs exceeding 100 kg [19]. Interpreting data obtained in pigs exceeding 100 kg is difficult and not comparable to the human setting. Thus, studies investigating the effects of cardioplegic solutions in pig models have good conditions for a high translation into the human setting. However, handling these animals for longer follow-up periods requires either the use of special requirements for pig husbandry, personnel staff, and institutional facilities.

## 4. Investigations in Adult Pig Models

A total of 42 studies reported the application of cardioplegic solutions in inducing cardiac arrest during cardiac surgery of adult pig models (Supplementary Table S1).

The St. Thomas-based cardioplegia was investigated in 27 studies (St. Thomas I: *n* = 7; St. Thomas II: *n* = 20), followed by 10 on blood cardioplegia, 9 on histidine-tryptophan-ketoglutarate (HTK)-based cardioplegia, and 8 that did not specify crystalloid cardioplegia. Cardioplegia induced by HTK-N (*n* = 2), Buckberg’s solution (*n* = 3), Del Nido (*n* = 1) and Braile (*n* = 1) were investigated to a lesser extent.

Cardioplegia studies aimed to identify the solution with the best properties for the human application. Therefore, direct comparisons of different cardioplegic solutions were performed. Comparisons between St. Thomas I and II cardioplegic solutions have shown enhanced functional recovery, better contractile efficiency, and improved energy status with St. Thomas I cardioplegia [21,22,23,24]. The novel HTK-N solution stabilizes hemoglobin and blood calcium levels, which can potentially increase kidney function [25]. Furthermore, HTK-N-induced cardioplegia resulted in fewer cerebral effects and inflammation during CPB surgery than HTK and appeared to exert protective effects in the brain [26]. The comparison of HTK and St. Thomas II cardioplegic solution showed better preservation of post-ischemic mechanoenergetic function and lower troponin T release with St. Thomas II-induced cardiopelgia [27]. Additives such as adenosine [28,29,30], pentazocine [28], lidocaine [28], procaine [29], cyclosporine A (CsA) [26,31], pyruvate [32,33], amrinone [34], cariporide [35], eniporide [36], aprotinin [37,38], nicorandil [39], H_2_S [40], zink-bis-histidinate [41], germinated brown rice extract (GBR) [42], and diazoxide [43] have been added to improve cardioplegic solutions. The addition of diaxozide, adenosine, and nicorandil to cardioplegic solutions preserved ventricular function [29,39,43,44]. Meanwhile, treatment with H_2_S, pyruvate, zink-bis-histidinate, or a combination of adenosine/lidocaine/pentazocine improved myocardial protection [33,40,41,42]. Pyruvate supplementation in cardioplegic solutions also decreased CPB-induced myocardial inflammation [32]. Aprotinin is able to reduce IRI and myocardial tissue edema, and preserve the vascular endothelial barrier [37,38]. A promoting effect on the coronary microcirculation was reported for a Mg^2+^-enriched crystalloid cardioplegic solution when compared with a potassium-enriched crystalloid cardioplegic solution [45]. GBR was reported to reduce the lactate production in CPB surgery [42]. The phosphodiesterase III inhibitor amrinone and CsA, which inhibits the mitochondrial permeability transition pore, promote cardiac function during cardioplegia [31,34]. While amrinone promotes rapid and sustained cardiac functional recovery by replenishing myocardial cyclic adenosine monophosphate [34], low-dose CsA supplementation enhanced basal mitochondrial respiration and preserved mitochondrial function, thereby diminishing the effects of IRI [31]. Inhibition of the Na^+^/H^+^ exchanger by eniporide and cariporide failed to show an effect on ventricular function or myocardial damage [35,36]. The majority of the investigations have been performed with an ischemic period ranging between 60–120 min (Supplementary Table S1) which correlates with the duration of ischemic periods in human surgery. Only four studies on St. Thomas cardioplegic solutions defined an ischemia duration of 30 min [30,46,47,48]. The on-pump reperfusion time varied between 10–180 min. A reperfusion period with a disconnection from CPB device, called off-pump reperfusion, ranged between 30–300 min.

## 5. Investigations in Pediatric Models

Cardioplegic solutions have been used in pediatric surgery. Thus, pediatric pig models were used to investigate the effects of the cardioplegic solutions. Sixteen studies reported the effects of different cardioplegic solutions, including St. Thomas cardioplegia (*n* = 9), HTK (*n* = 4), Del Nido (*n* = 2), and Calafiore/blood cardioplegia (*n* = 4) (Supplementary Table S2).

Direct comparisons between different cardioplegic solutions revealed that the modified Calafiore cardioplegia had a superior contractility after CBP surgery when compared to HTK [49]. For adult pig models, additives such as ebselene [50], olprinone [51], diazoxide [52], and sivelestat [53] have been investigated. A reduction in myocardial IRI with the antioxidants ebselene and olprinone has been proven [50,51]. Diazoxide protected the integrity of the mitochondrial structure when applied to a cardioplegic solution [52]. The neutrophil elastase inhibitor sivelestat reduced neutrophilic activation in the lungs and improved oxygenation after CPB in 7 to 14-week-old pigs [53]. The pediatric pig models investigated short-lasting ischemic periods of 10–45 min [54,55] as well as longer ischemic periods of 60–120 min (Supplementary Table S2). The on-pump and off-pump reperfusion periods ranged from 10 to 120 min and 30 min to 48 h, respectively.

## 6. Impact of Breeds, Strains, Age, and Sex

The species *Sus scrofa domestica* comprises several breeds that may vary in size and appearance, and can be classified into farm pigs and minipigs [17]. Farm pigs include breeds such as Yorkshire, Landrace, and Duroc. Minipig strains such as Yucatan, Göttingen, and Hanford are attractive due to low body weight at birth, early sexual maturity, and adult age. Their tissue properties are more mature and more resistant to surgical procedures [17]. The majority of investigations of cardioplegic solutions in infant and adult pig models have been performed in farm pigs. Only the groups of Sayk et al. [56] and Wu et al. [28] performed experimental investigations on minipigs. Farm pig breeds differ in their susceptibility to stress, growth rate, and fat content. In particular, susceptibility to stress during the preoperative period could influence the outcome of cardiovascular studies. Furthermore, their core body temperature and metabolism could differ slightly, which results in a bias on outcome parameters between different breeds. The age of the pigs had an indirect impact wherein the body weight of farm pigs rapidly increases with age. Consequently, the heart/body weight ratio decreased as described in the section on “Comparability of the heart anatomy and physiology in pig models and humans” and leads to alterations in basic cardiac physiology.

The impact of the sex of the pig on the outcome of cardioplegia-induced effects is unknown. Several studies have used pigs of both sexes to balance possible gender differences. However, there are also studies that exclusively used either male [40,41,49,57,58] or female pigs [21,48,59,60,61,62]. This can be influenced by additional experimental factors. For example, the withdrawal of urine in studies investigating kidney function during cardiac cardioplegia is easier in female pigs due to their anatomical features. Therefore, these investigations were performed only in female pigs. Thus, careful selection is necessary to determine the suitable breeds, strains, age, and sex for this study.

## 7. Refinement Strategies

Several aspects could be considered to refine preclinical investigations of cardioplegic solutions in pig models. Due to a special susceptibility to distress, it is necessary to avoid each conscious perceived stressful moment, such as a noise or any painful handling. Transportation to the operating room should be kept as short as possible. Intramuscular premedication consisting of midazolam, atropine, and ketamine is recommended.

To avoid the perception of any procedure-related pain, premedication of the sedated pigs (e.g., metamizole, fentanyl, or sufentanil) and anesthesia maintained by propofol and fentanyl, respectively, sufentanil is indicated.

Excessive hemodilution can have a significant impact on the outcome. Therefore, the total volume of the cardioplegic solution should be completely filtered. If blood donor pigs are included in the study, experiments should be planned such that the blood of one donor pig could be provided to several surgically-treated pigs. Furthermore, arterial and venous cannulas should be removed at the end of the CPB period, and the remaining blood in the tubes of the perfusion system should be re-transferred to the pig.

Intraischemic temperature could have a critical impact on the development of IRI. Therefore, monitoring of the cardiac temperature is recommended in the septum and left and right ventricles.

Sample withdrawal should comprise all organ systems that could be affected by IRI or supplements added to cardioplegic solutions. This allows for further investigation of this research field.

## 8. Limitations of Pig Models

Numerous limitations have been reported in studies investigating cardioplegic solutions for cardiac arrest. The restrictions relate to the small number of animals included in the studies, time limits, randomization and blinding, comparability with the clinical setting, study endpoints, and missing data and measurements (Table 1).

The most frequently mentioned restriction was the limited number of animals. In principle, the number of cases for an animal study should be determined according to the statistical calculation of the power and sample size, and is dependent on the primary and secondary endpoints of the study. Financial or human resources should not influence the sample size of the studies. Another limiting factor is the choice of the duration of the aortic cross-clamp, reperfusion, and recovery/observation period. A sufficiently long period of reperfusion is required for physiological weaning from CPB. However, pig models are known to deteriorate over time. In addition, statements about molecular changes in the organism can only be made with an appropriate duration of the reperfusion period, since some parameters requires hours to change. This led to the trend that parameters or markers are used for the analysis of cardioplegic effects that respond early and in a sensitive way to cardioplegia-induced ischemia or in the early reperfusion period (e.g., translocation of hypoxia-inducible factor 1α for oxidative stress or troponin T release into the blood). Additionally, a short reperfusion or observation period increased the risk that the study endpoints were not fully reached.

Implementation problems may arise when conducting the study in a blinded manner. For example, while experimental observers may be blinded to the study groups, it may be difficult to blind the surgeons or perfusionists when comparing crystalloid and blood cardioplegic solutions. However, blinding of the experimenters is strongly recommended to avoid biased results. Furthermore, simple or adaptive randomization is sufficient along with the learning curves of the surgical team (veterinarians, surgeons, and perfusionists).

Another key limitation is the use of young and healthy animals that do not have relevant clinical pathologies. Patients who undergo CPB surgery are usually older and have several comorbidities. Multimorbid patients may be more sensitive to CPB surgery. However, some of these effects can be reproduced in healthy animal models.

A further limitation results from the reduced oxygen transport capacity and hematocrit of pigs, leading to increased blood flow. This may result in a significantly stronger left ventricular wall, since it occurs in patients with pathological heart disease. The potassium serum concentration in healthy pigs ranges between 4.6 and 5.8 mmol/L [70]. According to Seutter et al., breed has no influence on potassium content in serum [71]. Despite the great similarity between pigs and humans, the results of these studies cannot be fully adapted to human medicine.

Particularly for pediatric applications, difficulties arise due to conflicts between newborn animal models and animal welfare [49,58]. Hence, this study aimed to investigate the following: (I) the choice of the appropriate animal model and duration of ischemia and reperfusion, (II) a detailed study planning including the consideration of all relevant factors and a statistical calculation of the sample size, and (III) a standardized operational process to ensure good reproducibility.

## 9. Conclusions

Porcine models for testing cardioplegic solutions in cardiac surgery have been used for the last 25 years, which generated information on cellular effects that could not be obtained from human trials. These investigations comprised results for cardioplegia in CPB procedures using adult and in infant porcine models in vivo. Different cardioplegic solutions have been compared or supplemented with drugs or additives that promote cell stability and protection to diminish the effects of IRI. Furthermore, the major limitations of pig models for investigating cardioplegic solutions are known. However, experimenters and preclinical investigator teams are encouraged to reduce these limitations within an experimental setting to achieve the best possible translation into the clinic.

## Figures and Tables

**Table 1:** Limitations in pig models for cardioplegic arrest.

Limitation	References
**Animal number**	
limited number of animals	[21,27,28,29,31,35,36,49,57,58,59,60,61,62,63,64]
**Time limits**	
short cross clamping time	[23,24,49,58]
short reperfusion/recovery time	[23,25,27,28,31,32,33,38,60,61,62,64,65,66]
short observation period/no long-term follow up	[26,32,59,63,65]
**Randomization & blinding**	
no randomization	[60,63]
surgeon/observer not blinded	[37,60,63]
**Comparability with clinical settings**	
use of young, healthy animals without clinically relevant pathology	[21,23,24,25,29,31,32,33,44,57,61,64]
results not fully comparable with humans	[26,29,44,49,58,67,68]
the use of neonatal piglets not allowed (animal protection requirements)	[49,58]
standardization of interventions/no individual treatment	[21,23,24,25,26,31,32,46,49,64]
model restricted to mild ischemia	[35,48,66]
reperfusion phase departed from clinical normality	[66]
**Study endpoints**	
effect on study endpoint not fully reached	[36,69]
endpoint not suited	[56,62]
lack of measurement of end-point related parameters	[26,50,68]
use of surrogate markers for endpoint measurement	[26]
**Missing data and measurements**	
missing control	[32,50]
the number of tested factors in one study limited	[24,57,65]
missing measurement/correlation with cardiac function	[56,61,69]
myocardial temperature not monitored	[55]
missing dose-response relationship for tested supplement	[50]
missing pressure-volume measurements	[21]
missing histological examination	[62]
wrong time point of blood/biopsy withdrawal	[44,60]

## Data Availability

No new data were created or analyzed in this study. Data sharing is not applicable to this article.

## Supplementary Materials

The following are available online at https://www.mdpi.com/article/10.3390/biomedicines9091279/s1, Table S1: Overview about investigations of cardioplegic solutions in adult pig models, Table S2: Overview about investigations of cardioplegic solutions in pediatric pig models.

## References

[1] Seyboldt-Epting W. (2013). Kardioplegie: Myokardschutz Während Extrakorporaler Zirkulation.

[2] Gravlee G.P. (2008). Cardiopulmonary Bypass: Principles and Practice.

[3] Donnelly A.J., Djuric M. (1991). Cardioplegia solutions. Am. J. Hosp. Pharm..

[4] Hoyer A., Kiefer P., Borger M. (2019). Cardioplegia and myocardial protection: Time for a reassessment?. J. Thorac. Dis..

[5] Ferguson Z.G., Yarborough D.E., Jarvis B.L., Sistino J.J. (2015). Evidence-based medicine and myocardial protection-where is the evidence?. Perfusion.

[6] Wegscheider K. (2016). Deutscher Herzbericht 2016.

[7] Holers V.M., Thurman J.M. (2004). The alternative pathway of complement in disease: Opportunities for therapeutic targeting. Mol. Immunol..

[8] Cavarocchi N.C., England M.D., Schaff H., Russo P., Orszulak T.A., Schnell W.A., O’Brien J.F., Pluth J.R. (1986). Oxygen free radical generation during cardiopulmonary bypass: Correlation with complement activation. Circulation.

[9] Janeway C.A., Travers P., Walport M., Shlomchik M. (2002). Immunologie.

[10] Cheluvappa R., Scowen P., Eri R. (2017). Ethics of animal research in human disease remediation, its institutional teaching; and alternatives to animal experimentation. Pharmacol. Res. Perspect..

[11] Hooijmans C.R., De Vries R., Leenaars M., Curfs J., Ritskes-Hoitinga M. (2011). Improving planning, design, reporting and scientific quality of animal experiments by using the Gold Standard Publication Checklist, in addition to the ARRIVE guidelines. Br. J. Pharmacol..

[12] Crick S.J., Sheppard M.N., Ho S.Y., Gebstein L., Anderson R.H. (1998). Anatomy of the pig heart: Comparisons with normal human cardiac structure. J. Anat..

[13] Nguyen P.K., Wu J.C. (2015). Large Animal Models of Ischemic Cardiomyopathy: Are They Enough to Bridge the Translational Gap?. J. Nucl. Cardiol..

[14] Leonhardt H. (1987). Anatomie des Menschen. Band II: Innere Organe.

[15] Sim E.K., Muskawad S., Lim C.-S., Yeo J.H., Lim K.H., Grignani R.T., Durrani A., Lau G., Duran C. (2003). Comparison of human and porcine aortic valves. Clin. Anat..

[16] Garg S., Singh P., Sharma A., Gupta G. (2013). A Gross Comparative Anatomical Study of Hearts in Human Cadavers and Pigs. Int. J. Med. Dent. Sci..

[17] Lelovas P.P., Kostomitsopoulos N., Xanthos T.T. (2014). A Comparative Anatomic and Physiologic Overview of the Porcine Heart. J. Am. Assoc. Lab. Anim. Sci..

[18] Hannon J.P., Bossone C.A., Wade C.E. (1990). Normal physiological values for conscious pigs used in biomedical research. Lab. Anim. Sci..

[19] Gallegos R.P., Rivard A.L., Bianco R.W. (2005). Animal models for cardiac research. Handbbok of Cardiac Anatomy, Physiology and Devices.

[20] Hiebl B., Mrowietz C., Ploetze K., Matschke K., Jung F. (2010). Critical hematocrit and oxygen partial pressure in the beating heart of pigs. Microvasc. Res..

[21] Santer D., Kramer A., Kiss A., Aumayr K., Hackl M., Heber S., Chambers D.J., Hallström S., Podesser B.K. (2019). St Thomas’ Hospital polarizing blood cardioplegia improves hemodynamic recovery in a porcine model of cardiopulmonary bypass. J. Thorac. Cardiovasc. Surg..

[22] Aass T., Stangeland L., Moen C.A., Solholm A., Dahle G.O., Chambers D.J., Urban M., Nesheim K., Haaverstad R., Matre K. (2019). Left ventricular dysfunction after two hours of polarizing or depolarizing cardioplegic arrest in a porcine model. Perfusion.

[23] Aass T., Stangeland L., Chambers D.J., Hallström S., Rossmann C., Podesser B.K., Urban M., Nesheim K., Haaverstad R., Matre K. (2017). Myocardial energy metabolism and ultrastructure with polarizing and depolarizing cardioplegia in a porcine model. Eur. J. Cardio-Thorac. Surg..

[24] Aass T., Stangeland L., Moen C.A., Salminen P.-R., Dahle G.O., Chambers D.J., Markou T., Eliassen F., Urban M., Haaverstad R. (2016). Myocardial function after polarizing versus depolarizing cardiac arrest with blood cardioplegia in a porcine model of cardiopulmonary bypass. Eur. J. Cardio-Thorac. Surg..

[25] Feirer N., Dieterlen M.T., Klaeske K., Kiefer P., Oßmann S., Salameh A., Borger M.A., Hoyer A. (2020). Impact of Custodiol-N cardioplegia on acute kidney injury after cardiopulmonary bypass. Clin. Exp. Pharmacol. Physiol..

[26] Hoyer A., Bergh F.T., Klaeske K., Lehmann S., Misfeld M., Borger M., Dieterlen M.T. (2019). Custodiol-N™ cardioplegia lowers cerebral inflammation and activation of hypoxia-inducible factor-1α. Interact. Cardiovasc. Thorac. Surg..

[27] Aarsaether E., Stenberg T.A., Jakobsen Ø., Busund R. (2009). Mechanoenergetic function and troponin T release following cardioplegic arrest induced by St Thomas’ and histidine-tryptophan-ketoglutarate cardioplegia‒an experimental comparative study in pigs. Interact. Cardiovasc. Thorac. Surg..

[28] Wu T., Dong P., Chen C., Yang J., Hou X. (2011). The myocardial protection of polarizing cardioplegia combined with delta-opioid receptor agonist in swine. Ann. Thorac. Surg..

[29] Jakobsen Ø., Muller S., Aarsæther E., Steensrud T., Sørlie D.G. (2007). Adenosine instead of supranormal potassium in cardioplegic solution improves cardioprotection. Eur. J. Cardio-Thorac. Surg..

[30] Vähäsilta T., Virtanen J., Saraste A., Luotolahti M., Pulkki K., Valtonen M., Voipio-Pulkki L.M., Savunen T. (2001). Adenosine in myocardial protection given through three windows of opportunity. An experimental study with pigs. Scand. Cardiovasc. J..

[31] Hoyer A.A., Klaeske K., Garnham J., Kiefer P., Salameh A., Witte K., Borger M., Dieterlen M.T. (2021). Cyclosporine A-enhanced cardioplegia preserves mitochondrial basal respiration after ischemic arrest. Perfusion.

[32] Ryou M.-G., Flaherty D.C., Hoxha B., Gurji H., Sun J., Hodge L.M., Olivencia-Yurvati A.H., Mallet R.T. (2010). Pyruvate-enriched cardioplegia suppresses cardiopulmonary bypass-induced myocardial inflammation. Ann. Thorac. Surg..

[33] Ryou M.-G., Flaherty D.C., Hoxha B., Sun J., Gurji H., Rodriguez S., Bell G., Olivencia-Yurvati A.H., Mallet R.T. (2009). Pyruvate-fortified cardioplegia evokes myocardial erythropoietin signaling in swine undergoing cardiopulmonary bypass. Am. J. Physiol. Circ. Physiol..

[34] Ko Y., Morita K., Nagahori R., Kinouchi K., Shinohara G., Kagawa H., Hashimoto K. (2009). Myocardial cyclic AMP augmentation with high-dose PDEIII inhibitor in terminal warm blood cardioplegia. Ann. Thorac. Cardiovas.c Surg..

[35] Bechtel J.F., Eichler W., Toerber K., Weidtmann B., Hernandez M., Klotz K.F., Sievers H.H., Bartels C. (2006). The Na+/H+ exchange inhibitor cariporide is washed out of the myocardium by crystalloid cardioplegia. Thorac. Cardiovasc. Surg..

[36] Klass O., Fischer U.M., Perez E., Easo J., Bosse M., Fischer J.H., Tossios P., Mehlhorn U. (2004). Effect of the Na+/H+ exchange inhibitor eniporide on cardiac performance and myocardial high energy phosphates in pigs subjected to cardioplegic arrest. Ann. Thorac. Surg..

[37] Khan T.A., Bianchi C., Araujo E., Voisine P., Xu S.H., Feng J., Li J., Sellke F.W. (2005). Aprotinin preserves cellular junctions and reduces myocardial edema after regional ischemia and cardioplegic arrest. Circulation.

[38] Khan T.A., Bianchi C., Voisine P., Feng J., Baker J., Hart M., Takahashi M., Stahl G., Sellke F.W. (2004). Reduction of myocardial reperfusion injury by aprotinin after regional ischemia and cardioplegic arrest. J. Thorac. Cardiovasc. Surg..

[39] Steensrud T., Nordhaug D., Elvenes O., Korvald C., Sørlie D. (2003). Superior myocardial protection with nicorandil cardioplegia. Eur. J. Cardio-Thorac. Surg..

[40] Osipov R.M., Robich M.P., Feng J., Chan V., Clements R.T., Deyo R.J., Szabo C., Sellke F.W. (2010). Effect of hydrogen sulfide on myocardial protection in the setting of cardioplegia and cardiopulmonary bypass. Interact. Cardiovasc. Thorac. Surg..

[41] Powell S.R., Nelson R.L., Finnerty J., Alexander D., Pottanat G., Kooker K., Schiff R.J., Moyse J., Teichberg S., Tortolani A.J. (1997). Zinc-bis-histidinate preserves cardiac function in a porcine model of cardioplegic arrest. Ann. Thorac. Surg..

[42] Demeekul K., Sukumolanan P., Bootcha R., Panprom C., Petchdee S. (2021). A Cardiac Protection of Germinated Brown Rice During Cardiopulmonary Bypass Surgery and Simulated Myocardial Ischemia. J. Inflamm. Res..

[43] Suarez-Pierre A., Lui C., Zhou X., Kearney S., Jones M., Wang J., Thomas R.P., Gaughan N., Metkus T.S., Brady M.B. (2020). Diazoxide preserves myocardial function in a swine model of hypothermic cardioplegic arrest and prolonged global ischemia. J. Thorac. Cardiovasc. Surg..

[44] Steensrud T., Nordhaug D., Husnes K.V., Aghajani E., Sørlie D.G. (2004). Replacing potassium with nicorandil in cold St. Thomas’ Hospital cardioplegia improves preservation of energetics and function in pig hearts. Ann. Thorac. Surg..

[45] Tofukuji M., Stamler A., Li J., Franklin A., Wang S.Y., Hariawala M.D., Sellke F.W. (1997). Effects of magnesium cardioplegia on regulation of the porcine coronary circulation. J. Surg. Res..

[46] Vähäsilta T., Saraste A., Kytö V., Malmberg M., Kiss J., Kentala E., Kallajoki M., Savunen T. (2005). Cardiomyocyte Apoptosis After Antegrade and Retrograde Cardioplegia. Ann. Thorac. Surg..

[47] Uotila P., Saraste A., Vähäsilta T., Kentala E., Savunen T. (2001). Stimulated expression of cyclooxygenase-2 in porcine heart after bypass circulation and cardioplegic arrest. Eur. J. Cardio-Thorac. Surg..

[48] Curro D., Bombardieri G., Barilaro C., Di Francesco P., Varano C., Possati G., Pragliola C. (1997). Time dependence of endothelium-mediated vasodilation by intermittent antegrade warm blood cardioplegia. Ann. Thorac. Surg..

[49] Münch F., Purbojo A., Kellermann S., Janssen C., Cesnjevar R.A., Rüffer A., Czerny M., Reser D., Eggebrecht H., Janata K. (2014). Improved contractility with tepid modified full blood cardioplegia compared with cold crystalloid cardioplegia in a piglet model. Eur. J. Cardio-Thorac. Surg..

[50] Chen Y., Liu J., Li S., Yan F., Xue Q., Wang H., Sun P., Long C. (2015). Histidine-Tryptophan-Ketoglutarate Solution with Added Ebselen Augments Myocardial Protection in Neonatal Porcine Hearts Undergoing Ischemia/Reperfusion. Artif. Organs.

[51] Kinouchi K., Morita K., Ko Y., Nagahori R., Shinohara G., Abe T., Hashimoto K. (2012). Reversal of oxidant-mediated biochemical injury and prompt functional recovery after prolonged single-dose crystalloid cardioplegic arrest in the infantile piglet heart by terminal warm-blood cardioplegia supplemented with phosphodiesterase III inhibitor. Gen. Thorac. Cardiovasc. Surg..

[52] Wang L., Kinnear C., Hammel J.M., Zhu W., Hua Z., Mi W., Caldarone C.A. (2006). Preservation of mitochondrial structure and function after cardioplegic arrest in the neonate using a selective mitochondrial KATP channel opener. Ann. Thorac. Surg..

[53] Ando M., Murai T., Takahashi Y. (2008). The effect of sivelestat sodium on post-cardiopulmonary bypass acute lung injury in a neonatal piglet model. Interact. Cardiovasc. Thorac. Surg..

[54] Liuba P., Johansson S., Pesonen E., Odermarsky M., Kornerup-Hansen A., Forslid A., Aburawi E.H., Higgins T., Birck M., Perez-de-Sa V. (2013). Coronary flow and reactivity, but not arrhythmia vulnerability, are affected by cardioplegia during cardiopulmonary bypass in piglets. J. Cardiothorac. Surg..

[55] Jones J., Wilson K., Koch W., Milano C. (2002). Adenoviral gene transfer to the heart during cardiopulmonary bypass: Effect of myocardial protection technique on transgene expression. Eur. J. Cardio-Thorac. Surg..

[56] Sayk F., Krüger S., Bechtel J.F., Feller A.C., Sievers H.H., Bartels C. (2004). Significant damage of the conduction system during cardioplegic arrest is due to necrosis not apoptosis. Eur J. Cardio-Thorac. Surg..

[57] Portilla-de Buen E., Leal C., Garcia-Martinez D., Cornejo A., Zepeda A., Aburto E. (2011). Pig heart preservation with antegrade intracellular crystalloid versus antegrade/retrograde miniplegia. J. Extra-Corpor. Technol..

[58] Janssen C., Kellermann S., Münch F., Purbojo A., Cesnjevar R.A., Rüffer A. (2015). Myocardial Protection During Aortic Arch Repair in a Piglet Model: Beating Heart Technique Compared With Crystalloid Cardioplegia. Ann. Thorac. Surg..

[59] Runge M., Hughes P., Gøtze J.P., Petersen R.H., Steinbrüchel D.A. (2006). Evaluation of myocardial metabolism with microdialysis after protection with cold blood- or cold crystalloid cardioplegia. A porcine model. Scand. Cardiovasc. J..

[60] Nakao M., Morita K., Shinohara G., Kunihara T. (2021). Modified Del Nido Cardioplegia and Its Evaluation in a Piglet Model. Semin. Thorac. Cardiovasc. Surg..

[61] Nakao M., Morita K., Shinohara G., Kunihara T. (2020). Excellent Restoration of Left Ventricular Compliance After Prolonged Del Nido Single-Dose Cardioplegia in an In Vivo Piglet Model. Semin. Thorac. Cardiovasc. Surg..

[62] Abe T., Morita K., Shinohara G., Hashimoto K., Nishikawa M. (2017). Synergistic effects of remote perconditioning with terminal blood cardioplegia in an in vivo piglet model. Eur. J. Cardio-Thorac. Surg..

[63] Nakao M., Morita K., Shinohara G., Saito S., Kunihara T. (2020). Superior restoration of left ventricular performance after prolonged single-dose del Nido cardioplegia in conjunction with terminal warm blood cardioplegic reperfusion. J. Thorac. Cardiovasc. Surg..

[64] Dahle G.O., Salminen P.-R., Moen C.A., Eliassen F., Jonassen A.K., Haaverstad R., Matre K., Grong K. (2015). Esmolol Added in Repeated, Cold, Oxygenated Blood Cardioplegia Improves Myocardial Function After Cardiopulmonary Bypass. J. Cardiothorac. Vasc. Anesthesia.

[65] Elvenes O.P., Korvald C., Myklebust R., Sørlie D. (2002). Warm retrograde blood cardioplegia saves more ischemic myocardium but may cause a functional impairment compared to cold crystalloid. Eur. J. Cardio-Thorac. Surg..

[66] Pathi V.L., McPhaden A.R., Morrison J., Belcher P.R., Fenner J.W., Martin W., McQuiston A.M., Wheatley D.J. (1997). The effects of cardioplegic arrest and reperfusion on the microvasculature of the heart. Eur J. Cardio-Thorac. Surg..

[67] Fannelop T., Dahle G.O., Salminen P.-R., Moen C.A., Matre K., Mongstad A., Eliassen F., Segadal L., Grong K. (2009). Multidose Cold Oxygenated Blood Is Superior to a Single Dose of Bretschneider HTK-Cardioplegia in the Pig. Ann. Thorac. Surg..

[68] Kajimoto M., Ledee D.R., Olson A.K., Isern N.G., Robillard-Frayne I., Des Rosiers C., Portman M.A. (2016). Selective cerebral perfusion prevents abnormalities in glutamate cycling and neuronal apoptosis in a model of infant deep hypothermic circulatory arrest and reperfusion. J. Cereb. Blood Flow Metab..

[69] Chen Y., Liu J., Li S., Li W., Yan F., Sun P., Wang H., Long C. (2013). Which is the better option during neonatal cardiopulmonary bypass: HTK solution or cold blood cardioplegia?. ASAIO J..

[70] Gürtler H., Neundorf R., Seidel H. (1987). Mittelwerte und Streuungsbereiche diagnostisch nutzbarer Parameter. Schweinekrankheiten.

[71] Seutter U. (1995). Einfluß von Rasse, Haltung, Fütterung, Management, Alter und Reproduktionsstadium auf hämatologische und Klinisch-chemische Parameter Beim Schwein.

