# Supplementary material for: Relevance and Recommendations for the Application of Cardioplegic Solutions in Cardiopulmonary Bypass Surgery in Pigs
Source: Biomedicines. 2021 Sep 21;9(9):1279. doi: 10.3390/biomedicines9091279 (PMC8464907; doi:10.3390/biomedicines9091279)
Supplement: Supplementary file 1 [file biomedicines-09-01279-s001.zip › biomedicines-1341674-supplementary.pdf]

biomedicines

MDPI

Supplemental Tables

Table S1. Overview about investigations of cardioplegic solutions in adult pig models.

Reference	Study Groups	Ischemia	Ischemic Temperature	On-pump Reperfusion	Off-pump Reperfusion	Cardioplegic Solution	Main Findings
Demeekul, 2021 [42]	*n* = 3 St. Thomas II cardioplegia<br>*n* = 3 St. Thomas II cardioplegia + GBR	20 min	32 °C	20-30 min	30 min	St. Thomas II	- GBR with cardioprotective effects against IRI<br>- GBR-supplemented cardioplegia induced lower lactate production level
Hoyer, 2021 [31]	*n* = 11 HTK cardioplegia<br>*n* = 11 HTK cardioplegia + CsA	90 min	34 °C	30 min	120 min	HTK;<br>HTK + CsA	- CsA supplementation enhanced the basal mitochondrial respiration thereby exerting a cardioprotective effect and diminishing IRI-induced damage<br>- CsA seems to preserve mitochondrial function via non-ROS related pathways
Nakao, 2020 [63]	*n* = 7 Del Nido cardiolegia + PlasmaLyte<br>*n* = 7 Del Nido cardioplegia	90 min	30 °C	30 min	30-60 min	Del Nido	- modified Del Nido cardioplegia comparable with the original Del Nido cardioplegia
Suarez-Pierre, 2020 [43]	*n* = 6 St. Thomas II cardioplegia<br>*n* = 6 St. Thomas II cardioplegia + diazoxide	120 min	32 °C	60 min	none	St. Thomas II	- diazoxide preserves systolic and diastolic ventricular function<br>- diazoxide allows safer prolonged ischemic times
Feirer, 2020 [25]	*n* = 10 HTK cardioplegia<br>*n* = 10 HTK-N cardioplegia	90 min	n.a.	120 min	none	HTK;<br>HTK-N	- HTK-N cardioplegia stabilized hemoglobin and Ca^{2+} levels → better kidney function

							- HTK-N with a trend for diminished AKI-related proximal tubule swelling and cytochrome c release
Santer, 2019 [21]	*n* = 6 St. Thomas I cardioplegia<br>*n* = 7 St. Thomas II cardioplegia	60 min	n.a.	60 min	120 min	St. Thomas I; St. Thomas II	- polarized cardiac arrest with similar myocardial protection and enhanced functional recovery
Hoyer, 2019 [26]	*n* = 10 HTK cardioplegia<br>*n* = 10 HTK-N cardiopelgia<br>*n* = 10 HTK cardioplegia + CsA	90 min	34 °C	120 min	none	HTK;<br>HTK-N;<br>HTK + CsA	- HTK-N with fewer cerebral effects and less inflammation during CPB surgery than HTK and HTK + CsA cardioplegia → HTK-N could exert brain protective effects
Aass, 2018 [22]	*n* = 10 St. Thomas I cardioplegia<br>*n* = 10 St. Thomas II cardioplegia	120 min	34 °C	10 min	240 min	St. Thomas I; St. Thomas II	- better myocardial contractile efficiency with polarizing cardioplegia (St. Thomas I) than with St. Thomas II
Aass, 2017 [23]	*n* = 10 St. Thomas I cardioplegia<br>*n* = 10 St. Thomas II cardioplegia	60 min	35 °C	10 min	180 min	St. Thomas I; St. Thomas II	- improved energy status and myocardial function with St. Thomas I cardioplegia than with St. Thomas II cardioplegia
Aass, 2016 [24]	*n* = 10 St. Thomas I cardioplegia<br>*n* = 10 St. Thomas II cardioplegia	60 min	34 °C	10 min	180 min	St. Thomas I; St. Thomas II	- comparable myocardial protection for St. Thomas I cardioplegia with esmolol, adenosine and Mg^{2+} and St. Thomas II<br>- improved LV contractile function with St. Thomas I cardioplegia
Dahle, 2015 [64]	*n* = 24 blood cardioplegia	100 min	35 °C	20 min	180 min	blood	- esmolol added to blood cardiopelgia preserved systolic cardiac function during the first 3 h after reperfusion

Portilla-de Buen, 2011 [57]	*n* = 4 HTK cardioplegia<br>*n* = 4 Braile miniplegia<br>*n* = 4 control (with CPB, w/o cardioplegia and ischemia)	60 min	36–38 °C	120 min	none	Braile (Miniplegia); HTK	- miniplegia and HTK cardioplegia with good myocardial protection<br>- Braile miniplegia with lower postreperfusion coronary vascular resistance, but with reduction of systemic concentrations of cardioplegic components<br>- HTK yielded higher K^+ and lower Na^{2+} blood concentrations
Wu, 2011 [28]	*n* = 5 St. Thomas II cardioplegia<br>*n* = 5 St. Thomas II cardioplegia + pentazocine<br>*n* = 5 St. Thomas II cardioplegia + adenosine + lidocaine<br>*n* = 5 St. Thomas II cardioplegia + adenosine + lidocaine + pentazocine	60 min	32–34 °C	20 min	120 min	St. Thomas II	- adenosin/lidocain/pentazocine cold crystalloid cardioplegia showed best myocardial protection and improved prolonged postoperative cardiac function
Osipov, 2010 [40]	*n* = 6 crystalloid cardioplegia + H_2S as infusion<br>*n* = 6 crystalloid cardioplegia + H_2S as bolus/infusion<br>*n* = 9 crystalloid cardioplegia + placebo	60 min	n.a.	n.a.	120 min	crystalloid cardioplegia	- H_2S treatment may offer myocardial protection via attenuation of caspase-independent apoptosis and autophagy
Ryou, 2010 [32]	*n* = 8 cardioplegia + pyruvate<br>*n* = 8 cardioplegia<br>*n* = 8 w/o cardiac arrest and cardioplegia	60 min	n.a.	30 min	240 min	4:1 blood:crystalloid solution	- pyruvate-enriched cardioplegia dampens CPB-induced myocardial inflammation<br>- increased GSH/GSSG and TIMP-2 mediate pyruvate's effects

Ko, 2009 [34]	*n* = 5 Buckberg's cardioplegia<br>*n* = 5 Buckberg's + amrinone cardioplegia<br>*n* = 5 St. Thomas II cardioplegia	90 min	normotherm	30 min	30 min	St. Thomas II; Buckberg	- high-dose amrinone with Buckberg's cardioplegia replenished myocardial cAMP and promoted rapid and sustained cardiac functional recovery
Ryou, 2009 [33]	*n* = 8 cardioplegia + pyruvate<br>*n* = 8 cardioplegia<br>*n* = 8 control (w/o cardiac arrest and cardioplegia)	60 min	n.a.	30 min	240 min	4:1 blood:crystalloid solution	- pyruvate induced EPO expression in the myocardium and activates EPO signaling<br>- intracoronary administration of pyruvate-enriched cardioplegia mobilized a cardioprotective mechanism
Aarsaether, 2009 [27]	*n* = 7 HTK cardioplegia<br>*n* = 7 St. Thomas II cardioplegia	60 min	n.a.	20 min	240 min	HTK;<br>St. Thomas II	- better preservation of postischemic mechanoenergetic function and lower TnT release with St. Thomas II than with HTK cardioplegia<br>- comparable LV function with St. Thomas II and HTK cardioplegia
Fanneloop, 2009 [67]	*n* = 8 single dose HTK cardioplegia<br>*n* = 8 repeated oxygenated blood cardioplegia	60 min	34 °C	20 min	180 min	HTK;<br>blood	- repeated oxygenated blood cardioplegia with better myocardial protection and LV function preservation than single dose HTK cardioplegia
Jacobson, 2007 [29]	*n* = 8 St. Thomas II cardioplegia<br>*n* = 8 adenosine-procaine-Mg^{2+} cardioplegia	60 min	n.a.	20–40 min	120 min	St. Thomas II; adenosine-procaine-Mg^{2+} solution	- adenosine instead of supranormal potassium in St. Thomas II cardioplegia gave satisfactory cardiac arrest, improved post cardioplegic LV systolic function and efficiency and attenuated myocardial cell damage
Runge, 2006 [59]	*n* = 6 blood:St. Thomas II cardioplegia + KCl	90 min	normotherm	n.a.	180 min	4:1 oxygenated blood:St. Thomas II	- cold blood cardioplegia with more rapid normalization of myocardial metabolism → superior cardiac protection

	n = 6 St. Thomas II cardioplegia						
Bechtel, 2006 [35]	*n* = 5 St. Thomas II cardioplegia + cariporide<br>*n* = 5 St. Thomas II cardioplegia + glucose (placebo)	60 min	32 °C	15 min	180 min	St. Thomas II	- no effect of i.v. cariporide on LV function or myocardial damage after cardioplegic arrest<br>- cariporide was washed out of the myocardium by repeated application of crystalloid cardioplegia
McCann, 2006 [72]	*n* = 10 4:1 modified Buckberg cardioplegia<br>*n* = 10 blood cardioplegia	90 min	normotherm	30 min	120 min	4:1 modified Buckberg; blood	- blood cardioplegia increased survival of hearts after weaning<br>- LV mass and myocardial edema lower after blood cardioplegia<br>- less volume necessary with whole blood cardioplegia
Vähäsilta, 2005 [46]	*n* = 8 antegrade modified St. Thomas II cardioplegia<br>*n* = 8 retrograde modified St. Thomas II cardioplegia	30 min	36 °C	30 min	30 min	modified St. Thomas II	- retrograde cardioplegia alone provided inferior cardioprotection against IRI in LV and RV
Khan, 2005 [37]	*n* = 7 crystalloid cardioplegia + aprotinin<br>*n* = 7 crystalloid cardioplegia	60 min	n.a.	10 min	90 min	crystalloid solution	- aprotinin preserved adherens junctions after regional ischemia and cardioplegic arrest via p38 MAPK pathway → preserved vascular endothelial barrier and reduced tissue edema in the myocardium
Klass, 2004 [36]	*n* = 7 eniporide before cardioplegia + added to HTK cardioplegia<br>*n* = 7 HTK cardioplegia with eniporide	60 min	28 °C	30 min	120 min	HTK	- no effects with the Na^+/H^+ exchange inhibitor eniporide on cardiac performance and high energy phosphate content

	n = 7 HTK cardioplegia w/o eniporide						
Sayk, 2004 [56]	*n* = 15 St. Thomas II cardioplegia<br>*n* = 7 Buckberg cardioplegia<br>*n* = 5 control (sternotomy)	60 min	32 °C	n.a.	180 min	St. Thomas II; Buckberg	- subendocardial Purkinje fibers much more vulnerable than working myocardium to IRI<br>- Purkinje fiber damage was due to necrosis rather than apoptosis
Steensrud, 2004 [44]	*n* = 8 modified St. Thomas I cardioplegia<br>*n* = 8 0.9% NaCl + nicorandil + Mg^{2+} + procaine cardioplegia	60 min	normotherm	20-40 min	120 min	modified St. Thomas I; 0.9% NaCl + nicorandil + Mg^{2+} + procaine	- better contractility and improved functional recovery with nicorandil-containing cardioplegia
Khan, 2004 [38]	*n* = 6 crystalloid cardioplegia + aprotinin<br>*n* = 6 crystalloid cardioplegia	60 min	n.a.	10 min	90 min	crystalloid solution	- aprotinin reduces IRI
Fischer, 2003 [73]	*n* = 13 HTK cardioplegia	60 min	28 °C	30 min	90 min	HTK	- cardioplegic arrest initiated apoptosis in myocardial epithelium and myocytes<br>- apotosis signal pathway activation not mediated by caspase-3 activation
Steensrud, 2003 [39]	*n* = 7 crystalloid cardioplegia<br>*n* = 7 blood cardioplegia<br>*n* = 7 blood cardioplegia + nicorandil	60 min	18 °C	20–40 min	120 min	crystalloid solution; hyperkalemic blood solution with and w/o nicorandil	- cold blood cardioplegia + nicorandil preserved LV contractility and myocardial energetics<br>- better diastolic function with cold blood cardioplegia + nicorandil
Elvenes, 2002 [65]	*n* = 7 cold St. Thomas II<br>*n* = 7 warm blood cardioplegia	60 min	32–37 °C	60 min	none	St. Thomas II	- St. Thomas II cardioplegia reduced the infarction size about 10% compared to control

	n = myocardial ischemia (control)						- warm blood cardioplegia reduced the infarction size by more than 50% compared to St. Thomas II cardioplegia<br>- functional cardiac impairment with both cardioplegic startegies during the first 60 min of reperfusion
Vähäsilta, 2001 [30]	*n* = 7 modified St. Thomas II cardioplegia + adenosine<br>*n* = 6 modified St. Thomas II cardioplegia	30 min	36 °C	90 min	none	modified St. Thomas II	- cardiomyocyte apoptosis involved in IRI<br>- no effects of adenosine addition to IRI
Uotila, 2001 [47]	*n* = 5 modified St. Thomas II cardioplegia<br>*n* = 5 sham operation<br>*n* = 6 control (no operation)	30 min	normotherm	90 min	none	modified St. Thomas II	- stimulation of COX-2 gene expression in the ventricular myocardium after CPB
Eising, 2000 [74]	*n* = 26 HTK cardioplegia	90 min	36.5–38.5 °C	30 min	300 min	HTK	- hemofiltration was ineffective in improving cardiac function or reducing the inflammatory response of CPB
Powell, 1997 [41]	*n* = 30 St. Thomas II cardioplegia with zinc-bis-histidinate<br>*n* = 30 St. Thomas II cardioplegia	60 min	28 °C	180 min	none	St. Thomas II	- zinc-bis-histidinate addition to cardioplegia was effective for myocardial preservation
Pathi, 1997 [66]	*n* = 6 30 min CPB<br>*n* = 6 30 min CPB + 90 min St. Thomas II cardioplegia<br>*n* = 6 30 min CPB + 90 min St. Thomas II cardioplegia + blood reperfusion	120 min	28 °C	30 min	none	St. Thomas II	- microvascular changes after CPB partially reversed by 30 min reperfusion<br>- leukocyte depletion did not ameliorate reversion processes

	n = 6 30 min CPB + 90 min St. Thomas II cardioplegia + leukocyte-depleted blood reperfusion						
Valen, 1997 [75]	*n* = 8 St. Thomas II cardioplegia	120 min	32–34 °C	30 min	none	St. Thomas II	- release of TnT, t-PA and histamin after 120 min cardioplegia<br>- different kinetics of TnT, t-PA and histamin release may indicate different affection of the myocardium and the endothelium
Wang, 1997 [76]	*n* = 6 crystalloid cardioplegia<br>*n* = 6 crystalloid cardioplegia + reperfusion<br>*n* = 6 blood cardioplegia<br>*n* = 6 blood + reperfusion<br>*n* = 6 sternotomy w/o cardioplegia	60 min	30 °C	15 min	45 min	crystalloid solution; blood	- comparable preservation of myocardial contractility or perfusion between crystalloid and blood cardioplegia
Tofukuji, 1997 [45]	*n* = 12 hyper-Mg crystalloid cardioplegia<br>*n* = 12 hyper-K crystalloid cardioplegia	60 min	n.a.	60 min	none	crystalloid solution	- hyper-Mg cardioplegia was superior to hyper-K cardioplegia in preserving coronary microcirculation
Irtun, 1997 [77]	*n* = 7 St. Thomas I cardioplegia at 75 mmHg<br>*n* = 7 St. Thomas I cardioplegia at 175 mmHg	120 min	26 °C	60 min	none	St. Thomas I	- high cardioplegic solution delivery pressure caused poorer postischemic recovery than moderate pressure<br>- cardioplegic solution delivery at 175 mmHg was harmful to pig heart
Curro, 1997 [48]	group 1: St. Thomas I cardioplegia	30–36 min	28 °C	30 min	none	St. Thomas I; 1:1 St. Thomas I : blood; blood	- intermittent warm blood cardioplegia superior to cold crystalloid cardioplegia regarding myocardial preservation

	group 2: 1:1 St. Thomas I : blood cardioplegia<br>group 3: blood cardioplegia						

Footnote Table S1: AKI, acute kidney injury; cAMP, cyclic adenosin monophosphate; Ca^{2+}, calcium; COX-2, cyclooxygenase-2; CPB, cardiopulmonary bypass; CsA, cyclosporine A; EPO, erythropeoietin; GBR, germinated brown rice; GSH, glutathione; GSSG, glutathione disulfide; HTK, histidin-tryptophan-ketoglutarat (Custodiol); HTK-N, histidin-tryptophan-ketoglutarat-N (Custodiol-N); H^+, hydrogen; H_2S, hydrogen sulfide; IRI, ischemia reperfusion injury; i.v., intravenous; K/K^+, potassium; KCl, potassium chloride; LV, left ventricle; Mg/Mg^{2+}, magnesium; $MgCl_2$, magnesium chloride; Na/Na^{2+}, sodium; RV, right ventricle; ROS, reactive oxygen species; TIMP-2, metalloproteinase-2; TnT, troponin T; t-PA, tissue plasminogen activator; w/o, without.

Table S2. Overview about investigations of cardioplegic solutions in pediatric pig models.

Reference	Study Groups	Ischemia	Ischemic Temperature	On-pump Reperfusion	Off-Pump Recovery	Cardioplegic Solution	Main Findings
Nakao, 2021 [60]	n = 7 Del Nido cardioplegia<br>n = 7 modified Del Nido cardioplegia	90 min	30 °C	30 min	40 min	Del Nido; modified Del Nido	- LV function and recovery after prolonged global ischemia comparable between original and modified Del Nido cardiopelgia
Nakao, 2020 [61]	n = 7 Del Nido cardioplegia with 90 min global ischemia<br>n = 7 Del Nido cardioplegia with 120 min global ischemia<br>n = 7 control with CPB only	90-120 min	30 °C	30 min	30 min	Del Nido	- excellent LV compliance after 120 min ischemia with Del Nido cardioplegia
Abe, 2017 [62]	n = 6 remote preconditioning<br>n = 6 terminal warm blood cardioplegia<br>n = 6 remote preconditioning + terminal warm blood cardioplegia<br>n = 6 simple aortic unclamp (control)	120 min	normotherm	30 min	30-60 min	St. Thomas II	- remote preconditioning with synergistic cardioprotection and LV functional recovery to warm blood cardioplegia
Kajimoto, 2016 [68]	n = 7 St. Thomas cardioplegia<br>n = 7 St. Thomas cardioplegia + selective cerebral perfusion	60 min	18 °C	45 min	60 min	St. Thomas	- selective cerebral perfusion prevents abnormalities in glutamate/glutamine/GABA cycling, which are induced by St. Thomas cardioplegia

Chen, 2015 [50]	n = 5 HTK cardioplegia<br>n = 5 HTK cardioplegia + ebselene<br>n = 5 CPB w/o cross clamping	120 min	30 °C	n.a.	120 min	HTK	- improved antioxidant defense, reduced myocyte apoptosis, better preserved mitochondrial structure, better myocardial protection with HTK + ebselene cardioplegia
Janssen, 2015 [58]	n = 10 HTK cardioplegia<br>n = 10 beating heart control	60 min	28 °C	120 min	6–8 h	HTK	- beating heart technique improved contractility<br>- comparable ischemic damage<br>- earlier rise of TnT levels after beating heart surgery<br>- more fluctuation of serum electrolytes with HTK cardioplegia
Münch, 2014 [49]	n = 8 modified Calafiore cardiopelgia<br>n = 12 HTK cardioplegia	60 min	28 °C	120 min	none	HTK;<br>modified Calafiore	- modified Calafiore cardioplegia with comparable myocardial protection as HTK cardiopelgia<br>- modified Calafiore cardioplegia with superior contractility post CPB
Chen, 2013 [69]	n = 7 single dose HTK cardiopelgia<br>n = 7 multi-dose St. Thomas cardioplegia<br>n = 7 CPB w/o cross clamping	120 min	25 °C	n.a.	180 min	HTK;<br>cold 1:1 St. Thomas/oxygenated blood	- HTK with sufficient cardiac protection for 2 hrs of ischemia<br>- HTK with equivalent myocardial protection to multidose cold blood cardioplegia
Liuba, 2013 [54]	n = 10 modified St. Thomas cardioplegia + CsA<br>n = 10 control w/o cardioplegic solution	45 min	32 °C	none	none	blood with modified St. Thomas 4:1	- St. Thomas cardioplegia associated with abnormalities in coronary vasomotor tone and receptor-related flow regulation<br>- no protective effect on coronary index or arrhythmia vulnerability after CPB with CsA-added cardioplegia

Kinouchi, 2012 [51]	n = 5 blood cardioplegia<br>n = 5 blood cardioplegia + olprinone<br>n = 5 uncontrolled reperfusion w/o blood cardioplegia	90 min	n.a.	30 min	30 min	blood	- blood cardioplegia + olprinone reduced myocardial reperfusion injury by reducing oxidant-mediated peroxidation<br>- blood cardioplegia accelerated prompt and persistent LV functional recovery with suppression of reperfusion arrhythmia
Shinohara, 2011 [78]	n = 6 St. Thomas II cardioplegia + 6 cycles unclamping/declamping of the aorta prior to reperfusion<br>n = 6 St. Thomas II cardioplegia + 10 cycles unclamping/declamping of the aorta prior to reperfusion<br>n = 6 St. Thomas II cardioplegia + simple removal of aorta crossclamp	90 min	n.a.	30 min	none	St. Thomas II	- postconditioning algorisms (unclamping and declamping cycles) promoted functional recovery after cardioplegic arrest
Ando, 2008 [53]	n = 7 St. Thomas II cardioplegia + sivelestat<br>n = 7 St. Thomas II cardioplegia	120 min	n.a.	n.a.	24 h	St. Thomas II	- intraoperative administration of sivelestat reduced neutrophil induction and activation in lung and improved oxygenation after CPB
Oka, 2008 [79]	n = 5 St. Thomas II cardioplegia<br>n = St. Thomas II cardioplegia + CsA pretreatment<br>n = 5 non-CPB control	60 min	28-30 °C	10–20 min	360 min	St. Thomas II	- CsA pretreatment prevented postcardioplegia alterations in mitochondrial structure and function
Wang, 2006 [52]	n = 5 St. Thomas II cardioplegia<br>n = 5 St. Thomas II cardioplegia + diazoxide	60 min	28-30 °C	10–20 min	6 h	St. Thomas II	- early apoptotic signaling events not prevented by diazoxide addition<br>- diazoxide added to St. Thomas II cardioplegic solution protected mitochondrial structure and functional integrity

Jones, 2002 [55]	n = 4 10 min cold crystalloid cardioplegia<br>n = 6 30 min cold crystalloid cardioplegia<br>n = 5 10 min warm crystalloid cardioplegia<br>n = 5 10 min warm circulatory arrest<br>n = 6 30 min cold crystalloid cardioplegia + Adeno-βGal	10–30 min	n.a.	n.a.	48 h	crystalloid cardioplegia	- adenoviral based gene transfer not impaired by lower temperatures associated with cold crystalloid cardioplegic arrest<br>- gene transfer is possible during cardiac surgery with CPB
Bolling, 1997 [80]	n = 5 blood cardioplegia<br>n = 5 St. Thomas II cardioplegia<br>n = 5 60 min hypoxia + blood cardioplegia<br>n = 5 60 min hypoxia + St. Thomas II cardioplegia	70 min	25 °C	30 min	6 h	blood;<br>St. Thomas II	- blood and St. Thomas II cardioplegia is cardioprotective and not compromised by preoperative hypoxia<br>- blood cardioplegia is superior to St. Thomas cardioplegia

Footnote Table S2: Adeno-βgal, β-galactosidase transgene; CPB, cardiopulmonary bypass; CsA, cyclosporine A; GABA, γ-aminobutyric acid; HTK, histidin-tryptophan-ketoglutarat (Custodiol); KCl, potassium chloride; LV, left ventricle; w/o, without.

References

72. McCann, U.G.; Lutz, C.J.; Picone, A.L.; Searles, B.; Gatto, L.A.; Dilip, K.A.; Nieman, G.F. Whole blood cardioplegia (minicardioplegia) reduces myocardial edema after ischemic injury and cardiopulmonary bypass. *J. Extra-Corpor. Technol.* **2006**, *38*, 14–21.
73. Fischer, U.M.; Klass, O.; Stock, U.; Easo, J.; Geissler, H.J.; Fischer, J.H.; Bloch, W.; Mehlhorn, U. Cardioplegic arrest induces apoptosis signal-pathway in myocardial endothelial cells and cardiac myocytes. *Eur. J. Cardio-Thorac. Surg.* **2003**, *23*, 984–990.
74. Eisng, G.P.; Schad, H.; Heimisch, W.; Gippner-Steppert, C.; Jochum, M.; Braun, S.L.; Mendler, N.; Meisner, H.; Lange, R. Effect of Cardiopulmonary Bypass and Hemofiltration on Plasma Cytokines and Protein Leakage in Pigs. *Thorac. Cardiovasc. Surg.* **2000**, *48*, 86–92.
75. Valen, G.; Sellei, P.; Owall, A.; Eriksson, E.; Kallner, A.; Waldum, H.; Risberg, B.; Vaage, J. Release of markers of myocardial and endothelial injury following cold cardioplegic arrest in pigs. *Scand. Cardiovasc. J.* **1997**, *31*, 45–50.
76. Wang, S.Y.; Stamler, A.; Tofukuji, M.; E Deuson, B.T.; Sellke, F.W. Effects of Blood and Crystalloid Cardioplegia on Adrenergic and Myogenic Vascular Mechanisms. *Ann. Thorac. Surg.* **1997**, *63*, 41–49.
77. Irtun, O.; Sørlie, D. High cardioplegic perfusion pressure entails reduced myocardial recovery. *Eur. J. Cardio-Thorac. Surg*. **1997**, *11*, 358–362.
78. Shinohara, G.; Morita, K.; Nagahori, R.; Koh, Y.; Kinouchi, K.; Abe, T.; Hashimoto, K. Ischemic postconditioning promotes left ventricular functional recovery after cardioplegic arrest in an in vivo piglet model of global ischemia reperfusion injury on cardiopulmonary bypass. *J. Thorac. Cardiovasc. Surg.* **2011**, *142*, 926–932.

79. Oka, N.; Wang, L.; Mi, W.; Zhu, W.; Honjo, O.; Caldarone, C.A. Cyclosporine A prevents apoptosis-related mitochondrial dysfunction after neonatal cardioplegic arrest. *J. Thorac. Cardiovasc. Surg*. **2008**, *135*, 123–130.
80. Bolling, K.; Kronon, M.; Allen, B.S.; Wang, T.; Ramon, S.; Feinberg, H. Myocardial protection in normal and hypoxically stressed neonatal hearts: The superiority of blood versus crystalloid cardioplegia. *J. Thorac. Cardiovasc. Surg.* **1997**, *113*, 994–1005.
